# Effect of higher currents on defibrillation success in out-of-hospital cardiac arrest: a multicentre cohort analysis

**DOI:** 10.1016/j.resplu.2025.101205

**Published:** 2025-12-23

**Authors:** Alexander Michael Lechleuthner, Kathrin Leupolz, Fabian Ripke, Sarah Dopfer, Jan Stock, Christian Miller, Yacin Keller, Marco Strohm

**Affiliations:** a City of Cologne Fire Department, Cologne, Germany; b GS Elektromedizinische Geräte G. Stemple GmbH, Kaufering, Germany; c L2R GmbH, Kürten-Herweg, Germany; d Emergency Department, University Hospital Leipzig, Leipzig, Germany; e City of Dresden Fire Department, Integrated Regional Control Centre, Dresden, Germany; f Institute for Security Science and Rescue Technology (ISR), Cologne, Germany

**Keywords:** Shock success, Energy, Impedance, Efficacy, Fibrillation, Waveform

## Abstract

**Background:**

Successful defibrillation requires sufficient electrical current to depolarize a critical mass of myocardial cells. The corpuls3 is available with the MAX option (MAX), which delivers its technically highest possible current for the actual impedance, instead of a fixed energy. This retrospective analysis compared shock success with MAX versus the standard 200 J setting.

**Methods:**

We retrospectively analysed ECG records from out-of-hospital cardiac arrests (OHCAs) in four German regions (May 2022–June 2024) to compare termination of fibrillation (TOF) and return of organised rhythm (ROOR) after shocks with MAX or 200 J.

**Results:**

A total of 1457 shocks with MAX and 2348 shocks with 200 J were included. First-shock success did not differ significantly between MAX and 200 J (TOF: 81 % vs. 76 %, *p* = 0.11; ROOR: 66 % vs. 64 %, *p* = 0.66). Considering all shocks, MAX achieved higher success rates (TOF: 80 % vs. 72 %, *p* < 0.001; ROOR: 67 % vs. 63 %, *p* = 0.02). When limiting the analysis to the first three shocks, patients treated exclusively with MAX achieved first TOF after 1.17 ± 0.44 shocks, compared to 1.36 ± 0.91 shocks for those receiving only 200 J (*p* = 0.02). Within three shocks, TOF was reached in 93.1 % of patients receiving only MAX versus 89.3 % receiving only 200 J (*p* = 0.05).

**Conclusion:**

MAX does not improve first-shock success compared to 200 J, but overall TOF and ROOR rates are significantly higher with MAX.

## Introduction

Ventricular fibrillation, a chaotic and life-threatening arrhythmia, necessitates prompt electrical defibrillation to restore organised cardiac rhythm and enable adequate hemodynamic function.^1^ Successful defibrillation depends on delivering sufficient electrical current to depolarize a critical mass of myocardial cells and terminate fibrillation.^2^ Modern biphasic defibrillators compensate for transthoracic impedance (TTI) by adjusting shock parameters in real time. However, patients with high TTI still receive less current than those with low TTI.^3^ Since mean current correlates with shock success,^4^, ^5^ patients with high-impedance may particularly benefit from higher mean current delivery.

The MAX variant of the corpuls3 offers the MAX option (MAX) in addition to standard discrete energy settings. Unlike defibrillation with a selected, discrete energy as the target value, MAX delivers the technically highest possible current for the actual impedance.

This study aimed to compare shock effectiveness between MAX and the standard 200 J setting in out-of-hospital cardiac arrest.

## Materials and methods

Post-Market Surveillance (PMS), as required by the European Medical Device Regulation (MDR), ensures continuous monitoring of medical device safety and performance after market release. Manufacturers must actively collect and analyse real-world data to confirm ongoing safety and effectiveness.

In this context, we conducted a pragmatic, multicentre, observational cohort study to monitor shock effectiveness with either 200 J or MAX during out-of-hospital cardiac arrest (OHCA). Defibrillators (MAX variant of corpuls3) with two default settings (200 J or MAX) were evenly distributed across emergency vehicles in four regions. The default setting for a particular resuscitation depended on the location and availability of the responding vehicle, resulting in random-like distribution of the two default settings.

Emergency personnel were trained and informed that they could deviate from the default setting at any time during resuscitation, as described in the device’s user instructions. Shock effectiveness was evaluated according to the actually chosen shock setting, not the default setting.

The ethics committee of the North Rhine-Westphalia Medical Association confirmed that, due to anonymized data and the non-interventional design, neither ethical approval nor consultation under §15 was required.

The current retrospective analysis aimed to evaluate the potential clinical benefits of higher mean currents delivered with MAX compared to 200 J.

### Defibrillator waveform and shock settings

The MAX variant of the corpuls3 (GS Elektromedizinische Geräte G. Stemple GmbH) is CE-certified and approved for routine clinical use. Like the standard corpuls3, it delivers impedance-compensated, rectilinear biphasic (RLB) shocks with fixed phase durations (6 ms first and 4 ms second). The key difference is the MAX option, which regulates current instead of energy and delivers the technically highest possible mean current for the actual impedance. To enable these higher currents, the MAX variant is equipped with a larger 100 µF capacitor. Across all impedances, MAX delivers higher mean current than defibrillation with 200 J, resulting in energy outputs up to 255 J ± 15 % (Table 1). For very low TTI (25 Ω), current is limited to prevent myocardial damage.

**Table 1:** Delivered energy stated in the user manual for different impedances for 200 J and MAX.

**Shock setting**	**Load**							**Accuracy**
	**25 Ω**	**50 Ω**	**75 Ω**	**100 Ω**	**125 Ω**	**150 Ω**	**175 Ω**	
200 J	151 J	197 J	198 J	188 J	190 J	197 J	194 J	±15 %
MAX	144 J	239 J	249 J	248 J	255 J	250 J	235 J	±15 %

### Study population and EMS system

The dataset included all patients with OHCA who received at least one defibrillation attempt with 200 J or MAX between May 2022 and June 2024 in four German EMS regions, regardless of presenting rhythm, age and the assumed aetiology of OHCA. The study centres (Mittelhessen, Bodensee-Oberschwaben, Cologne, Dresden) represent both urban and rural regions and operate a two-tier EMS system: paramedic-staffed ambulances and physician-staffed units dispatched for all OHCA cases. All emergency personnel were trained according to ERC Guidelines, allowing either fixed or escalating energy strategies for defibrillation. All centres regularly trained staff and used the corpuls3 as a standard device.

### Data collection and analysis

**Fig. 1:**
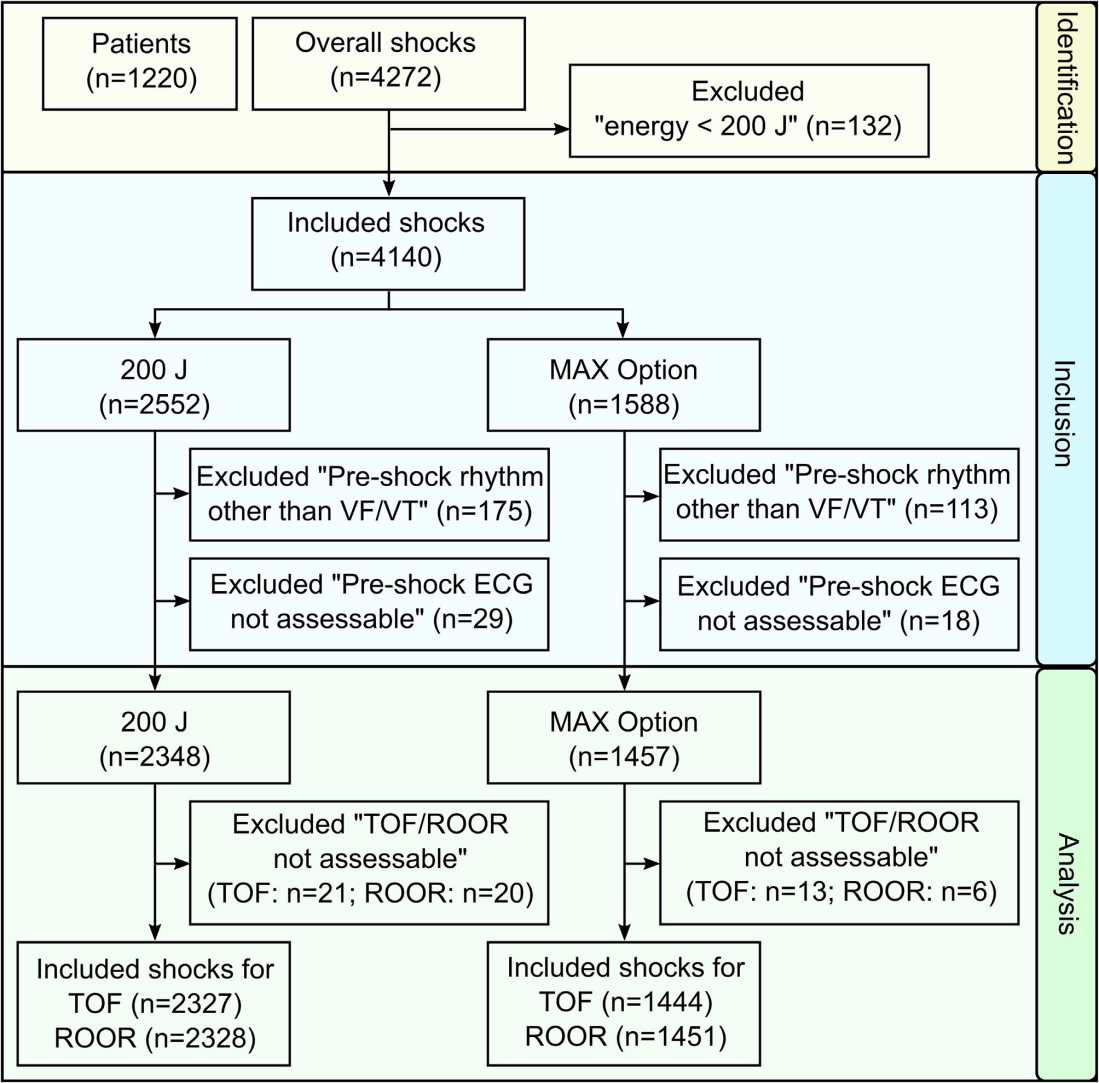
**Flowchart for inclusion and exclusion of data**.

Continuous ECG recordings and shock data were collected from device activation until the end of resuscitation, anonymized, and uploaded to a central database. ECGs were analysed by trained medical professionals and one emergency physician using a software tool that displayed the ECG before and after shock delivery and stored assessments of the pre-shock rhythm as well as TOF and ROOR. No additional filters were applied to reduce CPR artefacts, i.e. analysis could occur during chest compressions. Annotators were blinded to the shock setting (200 J or MAX). Shocks were excluded if ECG assessment was unreliable due to artefacts or if pre-shock rhythm was not ventricular fibrillation (VF) or ventricular tachycardia (VT) (Fig. 1).

### Outcome parameters

The primary outcome was termination of VF/VT (TOF) after the first shock, defined as either asystole or an organised rhythm at 5 s after shock delivery.

Secondary outcomes included overall TOF rate across all shocks, return of organised rhythm (ROOR) after the first shock and overall ROOR rate. ROOR was defined as at least two QRS complexes separated by no more than 5 s within 2 min after shock delivery.

To minimise potential bias from changes in shock settings during successive shocks, a subset analysis excluded patients who received both settings. In this subset, we assessed the number of shocks required for first TOF and the proportion of patients successfully defibrillated defined as achieving TOF within the first three shocks.

Additionally, shock-related parameters, such as transthoracic impedance before shock and mean current during the first phase were analysed.

### Statistical analysis

Descriptive statistics were expressed as mean ± standard deviations (SD) or median with interquartile range (IQR), as appropriate. For comparisons of TTI and mean current, either a *t*-test or a Mann-Whitney *U* test was applied depending on data distribution. Pearson’s chi-square test was used to compare proportions of patients with or without shock success. The relative risks (RR) with corresponding confidence intervals (CI) represent the ratio of the probability of TOF for MAX compared to 200 J. The significance level was set at *α* = 0.05. All analyses were performed using Python packages *statsmodels* and *SciPy*.

## Results

### Study population

Data from 1076 patients were analysed, including 2348 shocks with 200 J and 1457 shocks with the MAX option. The median number of shocks per patient was 3 (IQR: 1–5). Age and sex were documented in 22 % and 32 % of cases, respectively (Table 2). Within these limitations, mean age was 60 years and sex distribution was 73 % male and 27 % female.

**Table 2:** Study population characteristics. SD = standard deviation; IQR = interquartile range.

**Characteristics**		**Overall**		**200J**		**MAX**	
Sex, *n* (%)	Unknown	944	(88)	534	(88)	410	(88)
	Male	96	(73)	50	(68)	46	(78)
	Female	36	(27)	23	(32)	13	(22)
Information on age, *n* (%)		340	(32)	236	(22)	104	(10)
Age [years], mean (SD)		60 (±24)		58 (±25)		64 (±23)	
Impedance (1st shocks) [Ω], median (IQR)		91.0 (77.0–107.0)		93.5 (77.0–108.0)		90.5 (76.5–106.0)	
1st phases mean current [A], median (IQR)		18.0 (16.33–20.41)		17.1 (15.8–18.8)		20.5 (18.6–22.9)	

**Fig. 2:**
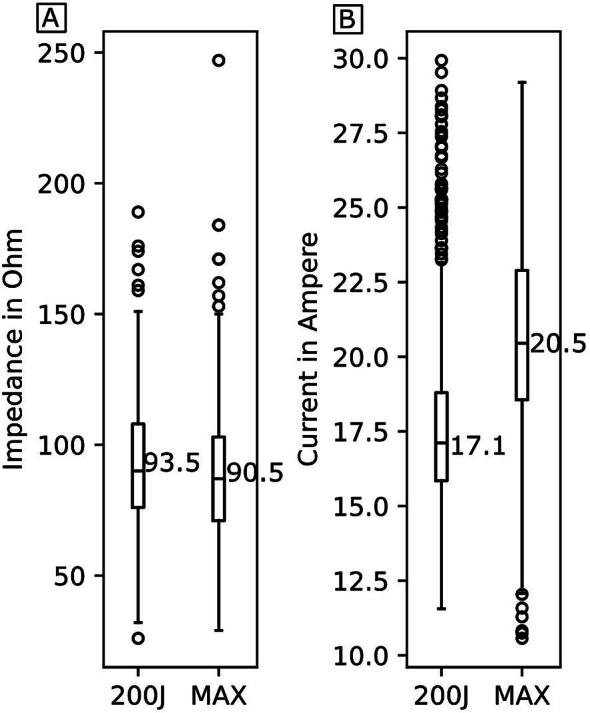
**Transthoracic impedance before first shock (A) and mean current of applied shocks (B)**.

There was no significant difference in transthoracic impedance (TTI) before the first shock between groups (200 J: 93.5 Ω vs. MAX: 90.5 Ω; *p* = 0.06; Fig. 2, Table 2). The median current of the first phase was significantly higher with MAX (20.5 A) compared to 200 J (17.1 A; *p* < 0.001; Fig. 2).

In 7 % (74/1076) of cases, the shock setting was changed during resuscitation. In 4 %, MAX was changed to 200 J at least once; in 1 %, 200 J was changed to MAX; and in 2 %, settings were changed more than once in either direction. In 26 % (19/74) of these cases, shock setting was changed after the preceding shock did not terminate VF/VT (from MAX to 200 J in 12.2 % (9 cases) and from 200 J to MAX in 13.5 % (10 cases)).

### Termination of fibrillation (TOF)

**Fig. 3:**
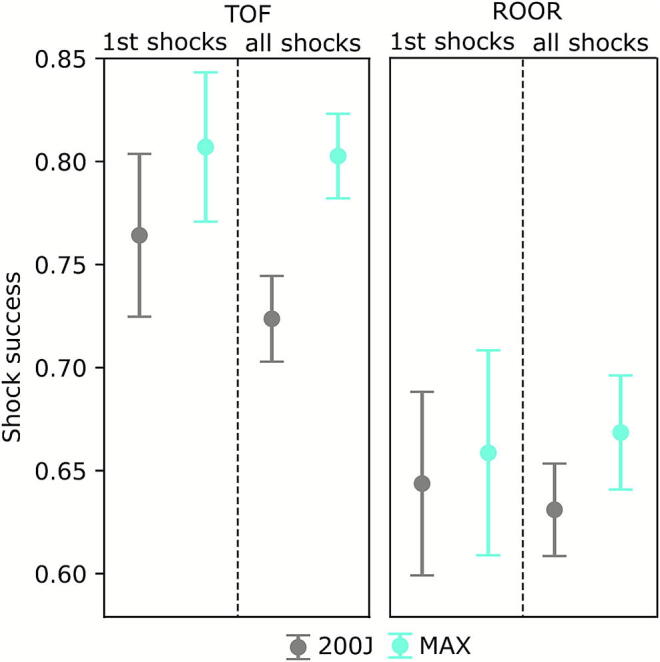
**TOF and ROOR rates of MAX and 200 J for the first shock and all shocks combined, respectively**.

First shocks terminated VF/VT in 81 % for MAX (95 %-CI: 77–84 %; *n* = 456) and 76 % for 200 J (95 %-CI: 73–80 %; *n* = 581; *p* = 0.11; Fig. 3). The RR for TOF after the first shock was 1.06 (95 %-CI: 0.99–1.13), indicating no significant advantage for MAX.

Considering all shocks combined, TOF rate was significantly higher with MAX (80 %; 95 %-CI: 78–82 %; *n* = 1444) compared to 200 J (72 %; 95 %-CI: 71–74 %; *n* = 2327; *p* < 0.001; Fig. 3). RR for overall TOF was 1.11 (95 %-CI: 1.07–1.15), indicating a higher likelihood with MAX compared to 200 J.

When limiting the analysis to the first three shocks, patients treated exclusively with MAX achieved first TOF after 1.17 ± 0.44 shocks, compared to 1.36 ± 0.91 shocks for those receiving only 200 J (p = 0.02). Within three shocks, at least one TOF was reached in 93.1 % (95 %-CI: 90.7–95.5 %) of MAX patients versus 89.3 % (95 %-CI: 86.8–91.8 %) with 200 J only (*p* = 0.05). RR for achieving TOF within three shocks was 1.04 (95 %-CI: 1.00–1.08), indicating a slight advantage for MAX.

### Return of organised rhythm (ROOR)

After the first shock, an organised rhythm returned in 66 % for MAX (95 %-CI: 62–70 %; *n* = 457) and 64 % for 200 J (95 %-CI: 60–68 %; *n* = 581; *p* = 0.66; Fig. 3). Considering all shocks, ROOR rate was significantly higher with MAX (67 %; 95 %-CI: 64–69 %; *n* = 1451) compared to 200 J (63 %; 95 %-CI: 61–65 %; *n* = 2328; *p* = 0.02; Fig. 3). RR for ROOR after the first shock was 1.18 (95 %-CI: 1.02–1.36), and RR across all shocks was 1.06 (95 %-CI: 1.01–1.11) indicating a modest benefit for MAX.

## Discussion

This study found no significant difference in first shock success rates – defined as termination of fibrillation (TOF) and return of organised rhythm (ROOR) – between MAX and 200 J. However, when all shocks delivered were considered, MAX demonstrated a significantly higher overall success rate for both TOF and ROOR compared with 200 J. The following sections address the clinical relevance and methodological considerations of these findings.

### Shock effectiveness

TOF reflects the electrical effectiveness of a defibrillation shock while minimising confounding from factors such as CPR quality or drug administration making it well suited to compare technical performance between shock settings. We defined TOF after the first shock as the primary outcome because it provides the most unbiased measure of shock effectiveness; later shocks are less comparable as success rates decline with increasing shock number^6^ and additional interventions may confound outcomes. However, we also analysed overall success across all shocks to reflect clinical reality. Many patients experience recurrent or persistent VF, requiring multiple shocks. Aggregating all shocks therefore captures real-world shock performance. Because multiple shocks per patient may not be statistically independent, we complemented the per-shock analysis with patient-level outcomes (proportion of patients defibrillated successfully and number of shocks required for success).

Unlike the selection of a discrete energy level (e.g., 200 J), MAX does not target a fixed energy but delivers the highest possible current for the actual impedance. Previous studies have shown that the selection of discrete energies is only partially suitable for dosing a defibrillation shock.^4^, ^5^ The decisive parameter for defibrillation, the transmyocardial current, varies considerably when a discrete energy is selected, depending on patient impedance,^7^ waveform and the type of impedance compensation.^3^ The concept of current-based defibrillation was investigated as early as 1996,^8^ demonstrating clinical feasibility with monophasic damped sine waveforms at currents of 25–40 A. Despite this, current-based defibrillation has not become standard practice and both, ERC and AHA Guidelines continue to recommend energy-based protocols.^9^, ^10^ Although optimal energy levels for defibrillation are unknown, ERC Guidelines recommend at least 120 J for the first shock with RLB waveforms, whereas for any subsequent shocks, an escalating or fixed energy protocol is acceptable. When MAX is selected, the delivered energy exceeds 120 J at all impedances, so it fulfils the requirements of the guidelines and can also be selected for the first shock.

Our results confirm previous findings that first shock success does not significantly differ between 120 J or 150 J, and 200 J^11^, ^12^ and suggest that this observation extends up to 260 J, as TOF and ROOR showed no significant difference between 200 J and MAX for the first shock. From a clinical perspective, the higher cumulative success rates with MAX may be relevant because most patients require multiple shocks, and faster termination of VF/VT may improve chances of ROSC and survival.^6^ The higher mean currents delivered with MAX likely explain this effect, as current is the decisive parameter for successful defibrillation.^3^

As expected, the higher currents compared to 200 J resulted in higher delivered energies with MAX (in mean 259 J vs. 196 J). It is unlikely that the higher currents and resulting higher energies resulted in significantly higher myocardial damage because mean current never exceeded 30 A and previous studies on different biphasic waveforms did not show myocardial damage even at maximum energy settings and repeated shocks.^13^, ^14^ Finally, our data suggest a potential benefit of applying higher currents.

### Comparison to previous studies

In this study, first shocks terminated VF/VT in 81 % (MAX) and 76 % (200 J). These rates are comparable to those reported by Spies et al., who, in a retrospective cohort study, reported a TOF rate of 77 % after the first shock with 200 J.^15^ In the literature, reported success rates vary considerably, ranging from approximately 67 %^16^ to values exceeding 80 %, and in some instances exceeding 90 %.^11^, ^17^, ^18^ Several factors may account for this divergence. First, some of these studies were conducted under more controlled or idealised conditions – for example, randomised controlled trials, as summarised by Morrison et al.^11^ – whereas our study was designed to reflect real-world clinical practice. Second, variations in patient characteristics, time to defibrillation or the rate of witnessed cardiac arrest likely contributed to the observed differences. In their observational study conducted in Rochester, USA, Hess et al. report a TOF rate of 92 % after the first shock with 84 % of these cardiac arrests being bystander-witnessed.^17^ Although the proportion of witnessed cardiac arrests is not explicitly documented in our dataset, our multicentre study – comprising four participating centres from the German EMS – reflects a representative population similar to that of the German Resuscitation Registry, which consistently reports a witnessed cardiac arrest rate of 58–68 %.^19^ Given the large sample size and the structural comparability, we refer to this published benchmark as a reasonable approximation. Furthermore, Hess et al. report a call-to-shock time of only 6.4 min, whereas the average call-to-shock time in Germany is 12.5 min.^20^ Given that witnessed cardiac arrest is a strong predictor of survival^21^ and that the probability of a successful shock decreases the longer ventricular fibrillation persists,^22^, ^23^ it is very likely that these factors contributed to the lower shock success rate in our dataset.

### Limitations

The data were collected within a non-interventional setting, within standard emergency care. The study design reflects real-world practice and regulatory requirements for post-market surveillance. Our pragmatic approach allowed inclusion of a large, representative cohort under routine conditions. However, the observational design introduces several limitations.

During the whole course of CPR, selection of 200 J or MAX was based predominantly on real-world clinical practice rather than adherence to an experimental protocol. This resulted in a different number of shocks being delivered at 200 J and MAX, respectively. It is unclear whether the even distribution of devices with different default settings (200 J or MAX) had an influence on the setting that was ultimately chosen. It is therefore also unclear whether the random-like allocation of devices with different default settings could reduce selection bias. In any case, the chosen approach is not equivalent to true randomisation, so confounding and selection bias are important limitations that must be taken into account when interpreting the results.

In addition, information on the circumstances of the cardiac arrest, time to first shock, pad position, and resuscitation measures preceding advanced life support (ALS) was unavailable. This includes interventions performed by first responders and bystanders, as well as the potential use of an automated external defibrillator (AED). These factors may have had an unequal influence on shock success rates. Moreover, the study did not control for potential confounding variables such as comorbidities or drug administration, which also might have influenced outcomes. Finally, information on age and sex was incomplete, preventing an accurate description of their distribution within the study cohort and the identification of paediatric patients.

Furthermore, the study was conducted in four regions within one country, which may limit generalizability to other healthcare systems or geographic settings.

Despite these limitations, the large, multicentre sample and its conduct within routine clinical practice enhance the external validity and generalizability of the findings. Future research should evaluate whether switching to MAX after an unsuccessful shock improves outcomes and assess the impact of MAX on clinically relevant endpoints such as ROSC and survival.

## Conclusion

In this large, multicentre observational study, first shock success did not differ between MAX and 200 J. However, MAX achieved a significantly higher overall success rate for both TOF and ROOR across all shocks. These findings suggest that higher mean current may enhance defibrillation effectiveness in real-world OHCA scenarios. However, these findings require confirmation in randomised controlled trials, ideally incorporating ROSC and survival.

## CRediT authorship contribution statement

**Alexander Michael Lechleuthner:** Writing – review & editing, Supervision, Conceptualization. **Kathrin Leupolz:** Writing – original draft, Visualization, Methodology, Formal analysis, Conceptualization. **Fabian Ripke:** Writing – original draft, Formal analysis. **Sarah Dopfer:** Writing – original draft, Methodology, Funding acquisition, Conceptualization. **Jan Stock:** Writing – review & editing, Project administration. **Christian Miller:** Writing – review & editing, Supervision, Funding acquisition. **Yacin Keller:** Writing – review & editing, Supervision, Funding acquisition. **Marco Strohm:** Writing – review & editing, Conceptualization.

## Declaration of competing interest

Kathrin Leupolz, Fabian Ripke and Sarah Dopfer are employees of GS Elektromedizinische Geräte G. Stemple GmbH and Jan Stock is executive officer of L2R GmbH. The financial relationship is limited to the monthly, contractually agreed salary. There is no additional financial or personal conflict of interest between the manufacturer and the authors. All other authors have nothing to disclose.
